# Coagulation factors: a novel class of endogenous host antimicrobial proteins against drug-resistant gram-negative bacteria

**DOI:** 10.1038/s41392-019-0083-4

**Published:** 2019-11-08

**Authors:** Congran Li, Xuefu You

**Affiliations:** Beijing Key Laboratory of Antimicrobial Agents, Institute of Medicinal Biotechnology, Chinese Academy of Medical Sciences & Peking Union Medical College, 100050 Beijing, China

**Keywords:** Drug screening, Target identification

In a recent study published in *Cell Research*, Dr. Xu Song’s group reported the potent antibacterial activity of three coagulation factors (VII, IX, and X) against gram-negative bacteria and hence discovered a novel class of endogenous host antimicrobial proteins.^1^

At present, antimicrobial resistance (AMR) poses significant challenges for clinical care and seriously threatens human health.^2,3^ Among the clinical pathogens, gram-negative bacteria are particularly problematic in terms of drug resistance because their lipopolysaccharide (LPS)-rich outer membrane reduces cellular permeability and acts as a target for antibacterial resistance development.^4^ Gram-negative bacterial infections have attracted worldwide concern in recent years, and new antibacterial drugs and novel therapeutic strategies that can address this healthcare issue are urgently needed.

Although many efforts have been made to develop novel antimicrobial agents, some endogenous host proteins with innate antibacterial activity may have been underappreciated. The coagulation factors VII, IX, and X are initiators of the clotting process; however, patients deficient in these factors were found to have bacterial infectious diseases (sepsis and pneumonia, for example) in addition to blood-clotting disorders.^5^ This finding leads to the assumption that the coagulation factors VII, IX, and X may have antibacterial function.

Research conducted by Song et al. showed that the coagulation factors VII, IX, and X possess antimicrobial activity against gram-negative bacteria, even extensively drug-resistant (XDR) pathogens, such as *Pseudomonas aeruginosa* and *Acinetobacter baumannii*.^1^ Both pathogens were recently listed among the 12 bacteria that pose the greatest threat to human health because of their antibiotic resistance by the World Health Organization.^6^

The coagulation factors VII, IX, and X are composed of two separate domains, i.e., a heavy chain (HC) and a light chain (LC). Their well-known role in the initiation of blood clotting is associated with the HC, which possesses serine protease activity.^7^ However, their antimicrobial activity revealed in this study is attributed to the LC. Using factor IX and its LC as an example, this study suggested that while serine protease activity of the factors is thermostable, their antibacterial activity is sensitive to heat treatment. Heat treatment is a major process for viral inactivation in manufacturing plasma-derived coagulation factor products,^8^ which may explain why the antibacterial activity was not observed for the widely used plasma-derived factors VII, IX, and X in patients with bleeding disorders.

**Fig. 1:**
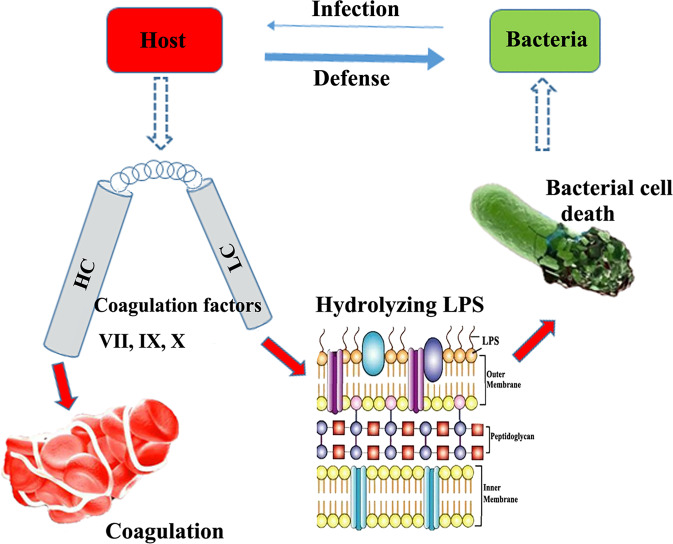
Antibacterial mechanism of the host coagulation factors VII, IX, and X against drug-resistant gram-negative bacteria

Examining the antibacterial mechanism further, the authors demonstrated that, unlike current antimicrobial agents that aim at cellular metabolism or the cytoplasmic membrane,^3,9^ the LC of the coagulation factors functions by hydrolyzing LPS in the bacterial outer membrane, leading to thorough damage of the bacterial morphology (Fig. 1). This unique mechanism renders bacteria less likely to develop resistance to the coagulation factors, and as LPS is present in almost all gram-negative bacteria, these factors should have general efficacy against gram-negative isolates, including XDR pathogens.^10^ Moreover, considering their origin from humans, these factors should have almost no toxicity toward mammalian cells.

Sepsis is a major reason for morbidity and mortality in bacterial infections, which is characterized by excessive and uncontrolled immune and coagulation responses, with LPS serving as a critical factor in the pathogenesis of gram-negative bacterial sepsis. Sensing of LPS by innate immune cells is vital for host defense against gram-negative bacteria, and dysregulation of the innate immune system (as seen in severe sepsis) will cause dramatic consequences for the infected host. No antimicrobial agents have been found to act by hydrolyzing LPS. Therefore, LPS hydrolysis triggered by the coagulation factors VII, IX, and X represents a new and promising strategy for antisepsis therapy.

The coagulation factors VII, IX, and X should have certain levels of antibacterial efficacy under physiological conditions, and in the case of injury, their recruitment to wounds will cause increased local concentrations and elevated antibacterial activities. Clotting disorders are associated with many diseases, such as stroke, and factors VII, IX, and X may act in the pathogenesis of these diseases by their functions in both blood clotting and anti-infection when massive bacterial infection occurs. Thus, a proper local concentration of these factors is critical for creating a balance between their positive and side effects.

Traditional antibacterial agents include antibiotics produced by microorganisms and semisynthetic derivatives or de novo-synthesized compounds. The seminal work of Song et al. identified a novel class of endogenous host antimicrobial proteins, the coagulation factors, which have broad prospects in clinical application and will also expand our knowledge of implying the coagulation system to host defense. However, there are still questions to be further explored, and these important results will stimulate researchers from different laboratories to produce exciting discoveries over the next few years.
